# Acute carbon monoxide poisoning with low saturation of carboxyhaemoglobin: a forensic retrospective study in Shanghai, China

**DOI:** 10.1038/s41598-021-97436-8

**Published:** 2021-09-17

**Authors:** Zheng Liu, Hang Meng, Juntian Huang, Pascal Kwangwari, Kaijun Ma, Bi Xiao, Liliang Li

**Affiliations:** 1 Department of Forensic Medicine, School of Basic Medical Sciences, Fudan University, 131 Dongan Road, Shanghai, 200032 China; 2 Shanghai Key Laboratory of Crime Scene Evidence, Shanghai Public Security Bureau, 803 North Zhongshan Road, Hongkou District, Shanghai, 200083 China

**Keywords:** Medical research, Risk factors

## Abstract

Carbon monoxide (CO) poisoning is a common cause of death, leading to morbidity and mortality worldwide. Features of the CO poisoning with low carboxyhemoglobin (COHb) levels remain to be characterized. This study collected a total of 307 CO poisoning cases from Shanghai Public Security Bureau, an official organization that handles the most complicated and life-threatening cases across Shanghai municipality in China, and regrouped these cases into three categories: group 1, 10% < COHb% < 30% (n = 58); group 2, 30% ≤ COHb% < 50% (n = 79); group 3, COHb% ≥ 50% (n = 170). Epidemiological, demographic, and forensic aspects of the CO poisoning cases, particularly those with low COHb levels, were analyzed. Our results showed that group 2 and 3 were mostly observed in younger victims (≤ 30 years), while group 1 equally distributed to all age groups (*p* = 0.03). All the CO poisoning from group 2 and 3 occurred in enclosed spaces, whereas cases from group 1 died additionally in outdoor spaces (*p* = 0.01). 81.03% of group 1 cases died in fire circumstances, while only 45.57% from group 2 and 30.59% from group 3 were fire-related (*p* = 0.00). Accordingly, group 1 was mostly related with fire burns, while group 2 or 3 were largely associated with gas leakage (*p* = 0.00). A combination with alcohol, but not other psychotropic drugs, associated with significant higher levels of blood COHb% in fire-unrelated (*p* = 0.021) but not fire-related cases (*p* = 0.23). Five extremely low COHb% (< 30%)-related poisoning deaths were negative of any cardiopulmonary pathology and psychoactive substances. In conclusion, CO poisoning with low COHb% significantly associates with fire circumstances and outdoor spaces and has no age preference. Further diagnostic markers mandates to be identified in order to avoid disputes in cases of extremely low COHb%-related poisoning.

## Introduction

Carbon monoxide (CO) is a colorless, odorless, non-irritant toxic gas which can cause harm to human body for its toxic effects ranging from cardiovascular and respiratory impairment to neuropsychiatric presentations and other acute complications^1,2^. CO is normally generated from the incomplete combustion of carbonaceous products^3^. Moreover, coal gas is still widely applied for residential heating and cooking^4^. Therefore, CO poisoning is the most common and plausible cause of fatal intoxication in circumstances of enclosed spaces (personal residents or public indoor spaces) or even outdoor places such as fire disasters.

The main CO poisoning mechanism is related to its combination with haemoglobin forming carboxyhaemoglobin (COHb), due to its higher affinity to haemoglobin, preventing the oxygen transportation and its use by cells and tissues. Once CO is inhaled, it binds with hemoglobin to form carboxyhemoglobin (COHb) with an affinity 200 times greater than oxygen that leads to decreased oxygen-carrying capacity, decreased release of oxygen to tissues and hence local hypoxia^5^. CO can also bind to myoglobin and cytochrome oxidases, and impairs oxygen consumption of skeletal muscles^6^. Acute CO poisoning always leads to hypotension and ischemia in the arterial border zones of the brain which accompany with loss of consciousness^5^.

To date, the diagnosis of acute CO poisoning is usually not difficult when the death circumstances corroborate an exposure to CO and toxicological analysis confirms the high saturation of COHb (COHb%). However, it could sometimes be challenging when the COHb% is not high enough (< 50%) or even at a lower value (< 30%) which contradicts with a clear history of CO exposure. Multiple explanations have been raised to address this contradiction. It has been suggested that decedents with potential cardiopulmonary pathology are more susceptible to CO, thus predisposing to death under a low COHb%^7^. The susceptibility of CO poisoning is also gender-dependent. Study has revealed female decedents, particularly during pregnancy, are more vulnerable to the CO gas and die with a lower COHb%^7,8^. The result of COHb% detection is also affected by lifestyle with smoking as one of the most documented factors. The baseline COHb% is relatively higher (~ 10%) in smokers, while it is usually less than 1% in non-smokers, making non-smoker group of decedents less tolerant to CO^9,10^. Alcohol and pharmaceutical drugs also play crucial roles in the blood COHb%. These substances might synergize with CO to promote cardiopulmonary repression^11^. Thus, multiple influential factors may affect the detected COHb%, making the interpretation of toxicological result challenging.

Multiple case serials have independently reported the existence of acute CO poisoning with low COHb% (refer to Table 1 for details). Ozturk et al. reported that a 50-year-old man was referred to the emergency room after exposure to fire smoke and his blood was detected with a COHb level of 20.70%^12^. In an intentional CO poisoning case, the blood COHb level of the victim was 19.30%^13^. Consistently, a recent analysis regrouped 3-year cases of suspected CO poisoning and identified low COHb%-related cases in the possible or highly probable CO poisoning groups^14^. In a retrospective study of 120 cases died from CO poisoning, 26 were detected to have a low COHb% (< 30%)^15^. A retrospective study from the National Institute of Legal Medicine and Forensic Sciences of Portugal, showed there was a case with cherry-red blood and viscera coloration but being only detected with a COHb saturation of 3%^16^. These reported cases were mostly at young ages and only approximate 20% (4 from the 18 cases) had clear cardiopulmonary pathology at autopsy (Table 1). This summary indicated that low COHb%-related acute CO poisoning might have specific characteristics. However, there is no study, to the best of our knowledge, that has systemically analyzed this type of cases.

**Table 1:** A selected summary of low COHb%-related CO poisoning in the case-report literatures.

Case No	Age, years	Gender	Medical history	Death location	CO source	COHb% (+ other substances if any)	Decomp	Ref
1	50	Male	AMI with stent for 3 yrs	NS	Fire smoke	20.70%	No	^12^
2	56	Female	HTN, DM and dyslipidemia	Private resident	Charcoal burning	19.30%	No	^13^
3	23	Female	Non-specific	4-Door sedan	Vehicle exhaust	44.90%	No	^17^
4	55	Male	ASCVD	an empty indoor pool	Power washing engine	27%	No	^18^
5	17	Male	NS	driver seat of a car	Exhaust emission	≤ 5%	No	^19^
6	NS	NS	NS	fire scene	Fire smoke	10%	No	^20^
7	NS	NS	NS	fire scene	Fire smoke	40%	No	^20^
8	41	Male	Repaired atrial septal defect	Rear seat of a car	CO gas tank	35.80% (alcohol 0.40 mg/mL)	No	^21^
9	41	Female	Non-specific	Rear seat of a car	CO gas tank	41.30% (alcohol 0.10 mg/mL)	No	^21^
10	23	Female	Non-specific	rear seat of a car	vehicle exhaust	21.63% (alcohol 2.13 mg/mL)	No	^22^
11	NS	Female	NS	Private resident	Gas leak	38.40%	Yes	^23^
12	40	Male	NS	Bedroom	Gas leak	20.00%	No	^24^
13	7	Male	NS	Bedroom	Gas leak	33.60%	No	^24^
14	5	male	NS	Bedroom	Gas leak	44.40%	No	^24^
15	NS	male	NS	dining room	Gas leak	42%	No	^25^
16	NS	Male	NS	Bedroom	Gas leak	38%	No	^25^
17	35	Male	Non-specific	Bedroom	Gas heater	23%	No	^26^
18	30	Male	Non-specific	Bedroom	Charcoal burning	40%	No	^26^

*NS* not specified, *AMI* acute myocardial infarction, *HTN* hypertension, *DM* diabetes mellitus, *ASCVD* atherosclerotic cardiovascular disease, *Decomp.* decomposed.

Since low COHb%-related poisoning is easily debated and disputed, the aim of this study is two-fold: (1) to analyze the epidemiology, demographic and toxicological features of all acute CO poisoning cases in Shanghai, China; and (2) to retrospectively analyze the features of CO poisoning with low COHb%. To obtain sufficient cases of low COHb%-related CO poisoning, we collected cases from the Shanghai Public Security Bureau (SPSB) which is an official organization that handles all the acute, complicated and life-threatening cases across the Shanghai municipality. This study intends to summarize the characteristics of CO poisoning with low COHb% and enhance the concept that more diagnostic methods are needed for the easily disputed cases.

## Materials and methods

### Setting

This study collected all the CO poisoning cases from the SPSB, Shanghai, China over the year 2000–2018. Shanghai is the largest metropolitan area in China which locates in the Eastern region and hosts residents over 30 million (about 2.5% of China’s overall population). The SPSB is an official organization that is responsible for conducting death investigations and certifying the cause and manner of most unnatural and unexplained deaths, particularly the complicated cases, in the 19 districts of the Municipality of Shanghai. The nature of the institute’s duty guaranteed collection of sufficient cases, particularly the low COHb%-related CO poisoning cases. The use of these cases for research purposes has been permitted by the Shanghai Key Laboratory of Crime Scene Evidence, SPSB, Shanghai, China.

The autopsy procedures/protocols at the SPSB have been consistent for the past decades. Each death was scene investigated at the time of case notification and autopsied when necessary within 24 h (usually within 2–4 h). All deaths investigated by the SPSB, which require medical examination, are subject to comprehensive toxicology detection for drugs and alcohol. For cases suspected of CO poisoning at notification, the replenished heart blood was fully filled into specific tubes and sent for dual-wavelength spectrophotometric testing as soon as possible to minimize any postmortem interference.

### Diagnostic criterion of acute CO poisoning

In the clinical practice, the diagnosis of acute CO poisoning is based on the elevated blood COHb%, the presence of clinical signs and symptoms after known exposure to CO. The degrees of CO poisoning have been described as mild poisoning (a COHb% of over 10% without clinical signs or symptoms of CO poisoning), moderate poisoning (a COHb% of over 10%, but under 20–25%, with minor clinical signs and symptoms of poisoning, such as headache, lethargy, or fatigue), and severe poisoning (a COHb% of over 20–25%, loss of consciousness, and confusion or signs of cardiac ischaemia, or both)^27^.

For the reasons that fatal poisoning cases might not have full records of clinical signs (i.e. acute deaths at home), we modified the clinical diagnostic criterion in a minor manner in accordance with forensic practice, namely (1) explainable source of environmental CO, (2) increased levels of COHb%, and (3) signs of CO poisoning. When all of the three points were met, it was diagnosed as acute CO poisoning. Each point of the forensic diagnostic criterion was documented in detail as below:

-The explainable source of CO: this was determined after identification of a CO source (i.e. a fire/explosion, a smell of gas, or a burning stove) and/or an increased concentration of CO at the scene environment. The normal concentrations of CO in enclosed spaces range from 0.5 to 5 ppm, which can increase to 15 ppm when being adjacent to gas stoves, to 5000 ppm in a home-lit fire and to 7000 ppm in an undiluted warm car exhaust without a catalytic converter^28^. Based on the *National Standard for Occupational Exposure Limits for Hazardous Agents in the Workplace* (standard ID: GBZ 2.1-2019), the occupational exposure limit of CO should be no greater than 24.0 ppm (30 mg/m^3^). When the environmental CO concentration exceeds the exposure limit, it is considered as increased CO concentration at the scene environment.

-Increased level of COHb%: Non-smokers living away from urban areas have COHb levels of 0.4–1.0%, reflecting endogenous carbon monoxide production, whereas levels of up to 5% may be considered normal in a busy urban or industrial setting^29^. Smokers are exposed to increased levels of carbon monoxide in cigarettes, and otherwise healthy heavy smokers can tolerate levels of COHb% of up to 15%^30^. A level of COHb% of over 10% in heart blood could be a vital index of CO poisoning. Therefore, for the purpose of this study, the increased level of COHb% was defined as heart blood COHb% of over 10%.

-Signs of CO poisoning: These signs include but not limit to cherry-red livor mortis, cyanosis of lips/fingernails, and muscle color turning into scarlet that are observed at medical examination.

### Inclusion criterion and case grouping

This study was a retrospective analysis of all the referred cases to SPSB within the abovementioned periods. All cases were diagnosed as acute CO poisoning by the SPSB after comprehensive analysis of scene investigation, medical examination findings, and toxicological results. In addition, the inclusion criteria for this study were: (1) Acute CO poisoning death without any medical interventions. (2) There were full data of scene investigation, medical examination records and toxicological report; (3) Corpse was not decomposed (no putrefaction, molded cadaver or skeletonized remains were observed); (4) Blood was immediately collected after notification of death and was sent for laboratory detection with hermetic seal. COHb% was immediately detected and results were accessed within 30 min. When other substances were negative or only considered as contributing factors to CO poisoning, these cases were considered to meet with the inclusion criterion.

Cases with fatal burning and/or fatal mechanical injury as the cause of death were excluded from this study, even though CO poisoning might be a contributing factor in fire-related circumstances. Cases that were recorded as questionable or uncertain CO poisoning deaths were also excluded. Cases with incomplete record of toxicology were excluded. This study only concentrated on cases with CO poisoning as the cause of death. Persistent CO poisoning death that refers to death with a survival time of over 24 h after CO poisoning, deaths from complications of CO poisoning like systemic sepsis and pneumonia were also excluded. Due to the rigorous criteria for case inclusion/exclusion, the direct causes of death for all the CO poisoning cases might be global asphyxia and ischemia. Other direct causes might not exist due to the short survival time of all cases. Therefore, all the included CO poisoning death cases were put together for analysis without detailed classification of direct causes of death.

For the purpose of this study, all cases were subgrouped as three categories based on the heart blood COHb%:

- Group 1, 10% < COHb% < 30%;
- Group 2, 30% ≤ COHb% < 50%;
- Group 3, COHb% ≥ 50%.

In this study, the statement of low COHb% refers to heart blood COHb% less than 50% (group 1 and group 2) and extremely low COHb% refers to that less than 30% (group 1).

### Information extraction and data analysis

For each case, epidemiology, demographic, scene investigation report and medical examination data including year, season, month, gender, age, examination record and toxicology results were extracted. In this study, spring defines March through May; Summer defines June through August; Autumn defines September through November, and Winter defines December through February. All data were tabulated in Microsoft Excel 2019, and figures were generated using the Graphpad Prism 8.0 (San Diego, CA, USA).

**Figure 1:**
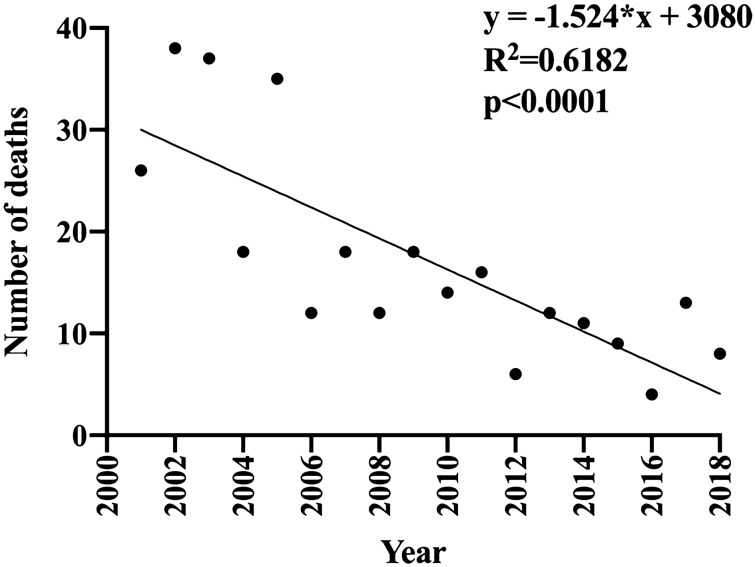
The time trend of CO poisoning cases in Shanghai, China. Linear regression analysis was performed and the correlation coefficiency was plotted.

Data were expressed as mean ± standard error of the mean or numbers (%) where the percentage (%) was calculated by dividing the number of a cell to the total number in the column. For comparisons between two groups, parametric Student’s *t*-test or nonparametric Mann–Whitney test was used. For ≥ 3 groups, one-way analysis of variance was used, followed by a Bonferroni post hoc test. The time trend in Fig. 1 was analyzed using liner regression analysis in Graphpad Prism 8.0. For categorical variables, the chi-squared test or Fisher’s exact test was used when necessary using SPSS 20.0 (IBM, Ehningen, Germany). A *p* value of less than 0.05 was considered as statistically significant.

## Results

### Overall distribution of CO poisoning cases

**Figure 2:**
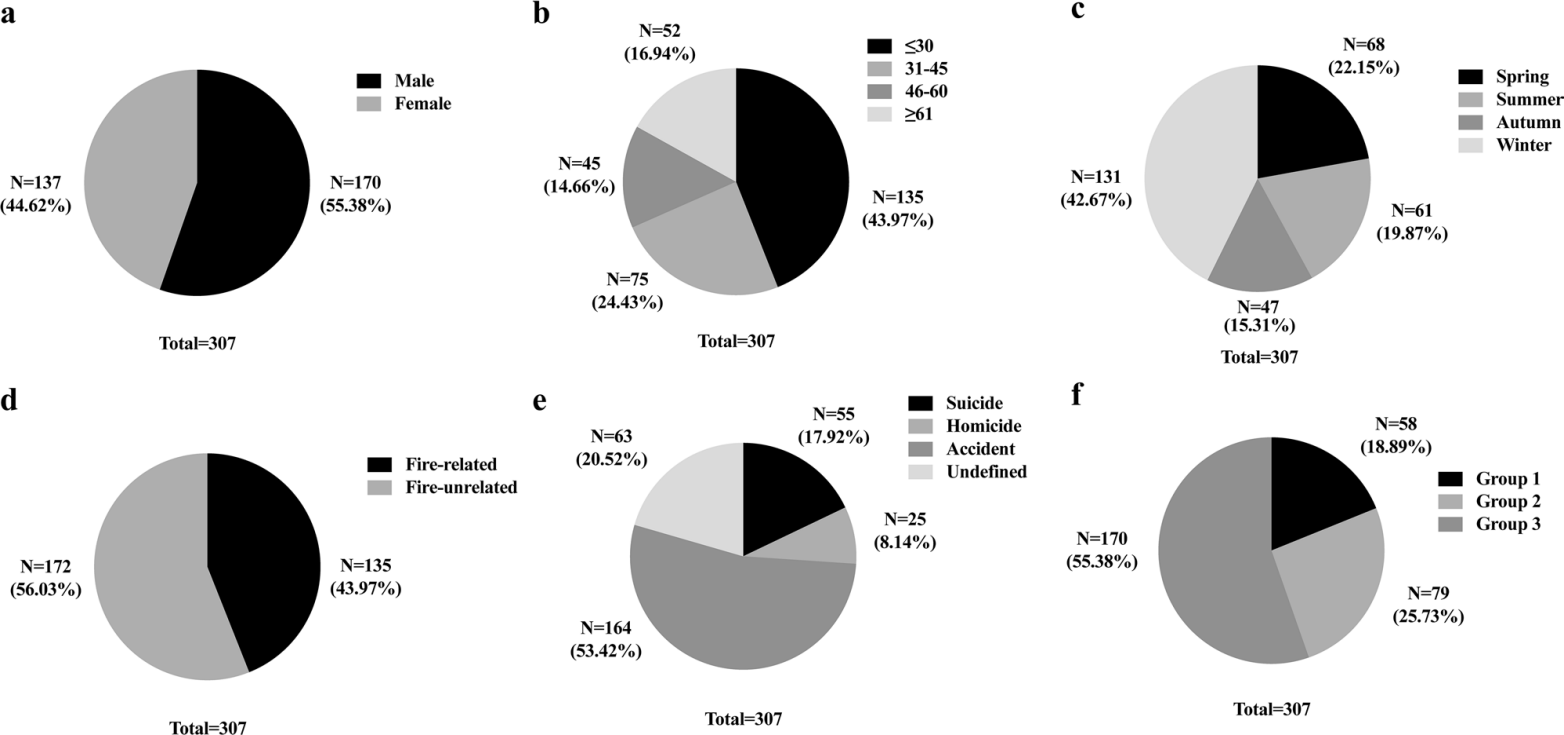
Distribution of the CO poisoning deaths by gender (**a**), age (**b**), season (**c**), fire-circumstance (**d**), manner of death (**e**), and heart blood COHb% (**f**). Group 1: 10 < COHb < 30% (n = 58); group 2, 30% ≤ COHb% < 50% (n = 79); group 3, COHb% ≥ 50% (n = 170).

During the studied periods, a total of 307 deaths were due to acute CO poisoning. The number of deaths due to CO poisoning significantly decreased over years (correlation coefficiency = 0.6182; regression *p* < 0.0001) (Fig. 1). Male victims were more common (n = 170, 55.38%) with a male/female ratio as 1.24:1 (Fig. 2a). Victims at the age younger than 30 years accounted for 135 cases (43.97%), followed by those at 31–45 years (n = 75 cases, 22.43%), at ≥ 61 years (n = 52 cases, 16.94%), and at 46–60 years (n = 45 cases, 14.66%) (Fig. 2b). Winter claimed 42.67% of deaths (n = 131), followed by Spring (n = 68 cases, 22.15%), Summer (n = 61 cases, 19.87%), and Autumn (n = 47 cases, 15.31%) (Fig. 2c). A total of 135 cases (43.97%) were fire-related, while the left 172 cases (56.03%) were fire-unrelated (Fig. 2d). Over half of the cases (n = 164, 53.42%) were accidental. Fifty-five cases (17.92%) were suicidal and 25 cases (8.14%) were homicidal. The remaining 63 cases (20.52%) were undetermined with regard to the manner of death (Fig. 2e).

We then divided all the CO poisoning cases into 3 subgroups based on the heart blood COHb%. It turned out that 58 of the 307 cases (18.89%) were assigned to group 1. Seventy-nine (25.73%) were from group 2 and 170 cases (55.38%) were from group 3 (Fig. 2f).

### Distribution of each subgroup of cases by epidemiologic and demographic parameters

The incidence of group 2 and group 3 cases linearly decreased with years (*p* = 0.017, Table 2). The incidence of group 1 cases did not linearly decrease but remained relatively high (~ 20%) in recent years (Table 2). The number of CO poisoning cases from the above groups did not differ between gender (*p* = 0.42) and among the manners of death (*p* = 0.06) but significantly differ among age groups (*p* = 0.03) and seasons (*p* = 0.002) (Table 2). All of the 3 groups showed preference occurring in winter (*p* = 0.002). Group 1 of cases were mainly evenly distributed among all age groups but largely occurred in the Winter (50.00%), whereas group 2 and group 3 of cases were mainly concentrated in the 0–30 years as well as the 31–45 years and distributed to each season (Table 2).

**Table 2:** Distribution of CO poisoning cases by epidemiologic and demographic categories.

Category	Group 1, n = 58	Group 2, n = 79	Group 3, n = 170	*p* value
Year				0.02
2001–2006	28 (48.28%)	53 (67.09%)	85 (50.00%)	
2007–2012	13 (22.41%)	18 (22.78%)	53 (31.18%)	
2013–2018	17 (29.31%)	8 (10.13%)	32 (18.82%)	
Gender				0.42
Male	36 (62.07%)	40 (50.63%)	94 (55.29%)	
Female	22 (37.93%)	39 (49.37%)	76 (44.71%)	
Age, years				0.03
≤ 30	16 (27.59%)	33 (41.77%)	86 (50.59%)	
31–45	16 (27.59%)	20 (25.32%)	39 (22.94%)	
46–60	9 (15.52%)	15 (18.99%)	21 (12.35%)	
≥ 61	17 (29.31%)	11 (13.92%)	24 (14.12%)	
Season				0.002
Spring	10 (17.24%)	22 (27.85%)	36 (21.18%)	
Summer	9 (15.52%)	9 (11.39%)	43 (25.29%)	
Autumn	10 (17.24%)	21 (26.58%)	16 (9.41%)	
Winter	29 (50.00%)	27 (34.18%)	75 (44.12%)	
Manner of death				0.06
Suicide	11 (18.97%)	6 (7.59%)	38 (22.35%)	
Homicide	4 (6.90%)	6 (7.59%)	15 (8.82%)	
Accident	28 (48.28%)	53 (67.09%)	83 (48.82%)	
Undefined	15 (25.86%)	14 (17.72%)	34 (20.00%)	

### Distribution of each subgroup of cases by circumstantial parameters

Cases from both group 2 and group 3 were all occurred in indoor places (private residence or public indoors) but there was a minimal proportion of decedents (3.45%) died in outdoor places in group 1 (*p* = 0.01, Table 3). A lower COHb% was more likely to be detected in fire burn-related cases (*p* = 0.00), as group 1 composed of as high as 81.03% fire burn-related cases. Consistently, fire was the most common source for group 1 of cases (74.14%), while gas leakage constituted the vast majority of cases from group 3 (62.35%). Explosion gas and bathroom gas heater also claimed sparse deaths. Vehicle exhaust caused 5 cases of CO poisoning which were all detected with high COHb% in group 3 (Table 3).

**Table 3:** Distribution of the CO poisoning cases by death circumstances.

Category	Group 1, n = 58	Group 2, n = 79	Group 3, n = 170	*p* value
Location				0.01
Private residence	47 (81.03%)	58 (73.42%)	146 (85.88%)	
Public indoor	9 (15.52%)	21 (26.58%)	24 (14.12%)	
Public outdoor	2 (3.45%)	0 (0.00%)	0 (0.00%)	
Fire-related*				0.00
Yes	47 (81.03%)	36 (45.57%)	52 (30.59%)	
No	11 (18.97%)	43 (54.43%)	118 (69.41%)	
Source				0.00
Gas leakage	9 (15.52%)	38 (48.10%)	106 (62.35%)	
Fire	43 (74.14%)	36 (45.57%)	54 (31.76%)	
Explosion	4 (6.90%)	1 (1.27%)	3 (1.76%)	
Bathroom gas heater	2 (3.45%)	4 (5.06%)	2 (1.17%)	
Vehicle exhaust	0 (0.00%)	0 (0.00%)	5 (2.94%)	

*Defines the cases with acute CO poisoning as the cause of death in circumstances of fire burn incidences.

### Distribution of each subgroup of cases by toxicological results

To analyze the contributing drugs, the toxicological results were stratified as mere CO, CO plus alcohol, and CO plus psychotropic drugs. It was found that the 3 groups of cases did not significantly differ when all the CO poisoning cases were pooled together (inclusion of fire burn cases) (*p* = 0.23, Table 4). However, when fire burn-related cases were excluded (case number = 172), the 3 groups of cases were significantly different with regard to toxicology (*p* = 0.021). The group 1 of cases were all detected with mere CO, while several cases from group 2 and substantial cases from group 3 were combined with alcohol or psychotropic drugs (*p* = 0.021, Table 5).

**Table 4:** Distribution of CO poisoning deaths by toxicology with inclusion of fire-related cases (n = 307).

Toxicology	Group 1, n = 58	Group 2, n = 79^a^	Group 3, n = 170^b^	*p* value
Mere CO	47 (81.00%)	70 (88.61%)	136 (80.00%)	0.23
CO plus alcohol	10 (17.20%)	6 (7.59%)	27 (15.90%)	
CO plus psychotropic drugs	1 (1.70%)	4 (5.06%)	11 (6.50%)	

^a^1 Case with combined alcohol and psychotropic drugs were repeatedly allocated to each category.

^b^4 Cases with combined alcohol and psychotropic drugs were repeatedly allocated to each category.

**Table 5:** Distribution of CO poisoning deaths by toxicology after exclusion of fire–related cases (n = 172).

Toxicology	Group 1, n = 11	Group 2, n = 43^a^	Group 3, n = 118^b^	*p* value
Mere CO	11 (100.0%)	40 (93.02%)	89 (75.42%)	0.021
CO plus alcohol	0 (0.0%)	1 (2.33%)	23 (19.49%)	
CO plus psychotropic drugs	0 (0.0%)	3 (6.98%)	10 (8.47%)	

^a^1 Case with combined alcohol and psychotropic drugs were repeatedly allocated to each category.

^b^4 Cases with combined alcohol and psychotropic drugs were repeatedly allocated to each category.

**Figure 3:**
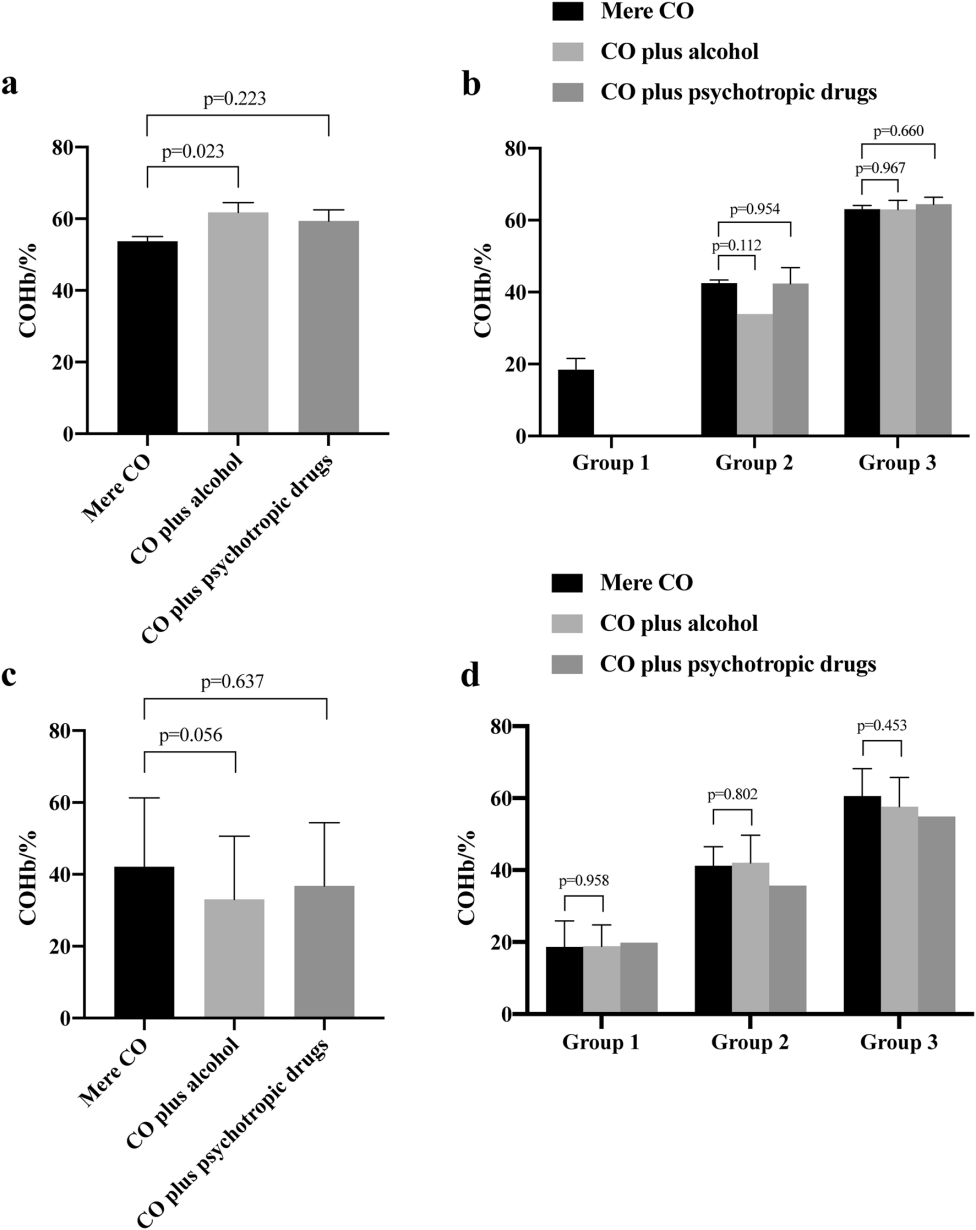
Distribution of COHb saturation by toxicological results in decedents died from CO poisoning. (**a**, **b**) In the fire-unrelated CO poisoning cases (n = 174), the distribution of COHb saturation by toxicology in all the cases (**a**) or in each subgroup (**b**) was presented. (**c**, **d**) In the fire-related CO poisoning cases, the distribution of COHb saturation by toxicology in the pooled cases (**c**) or in each subgroup (**d**) was presented. *p* value was as indicated.

**Figure 4:**
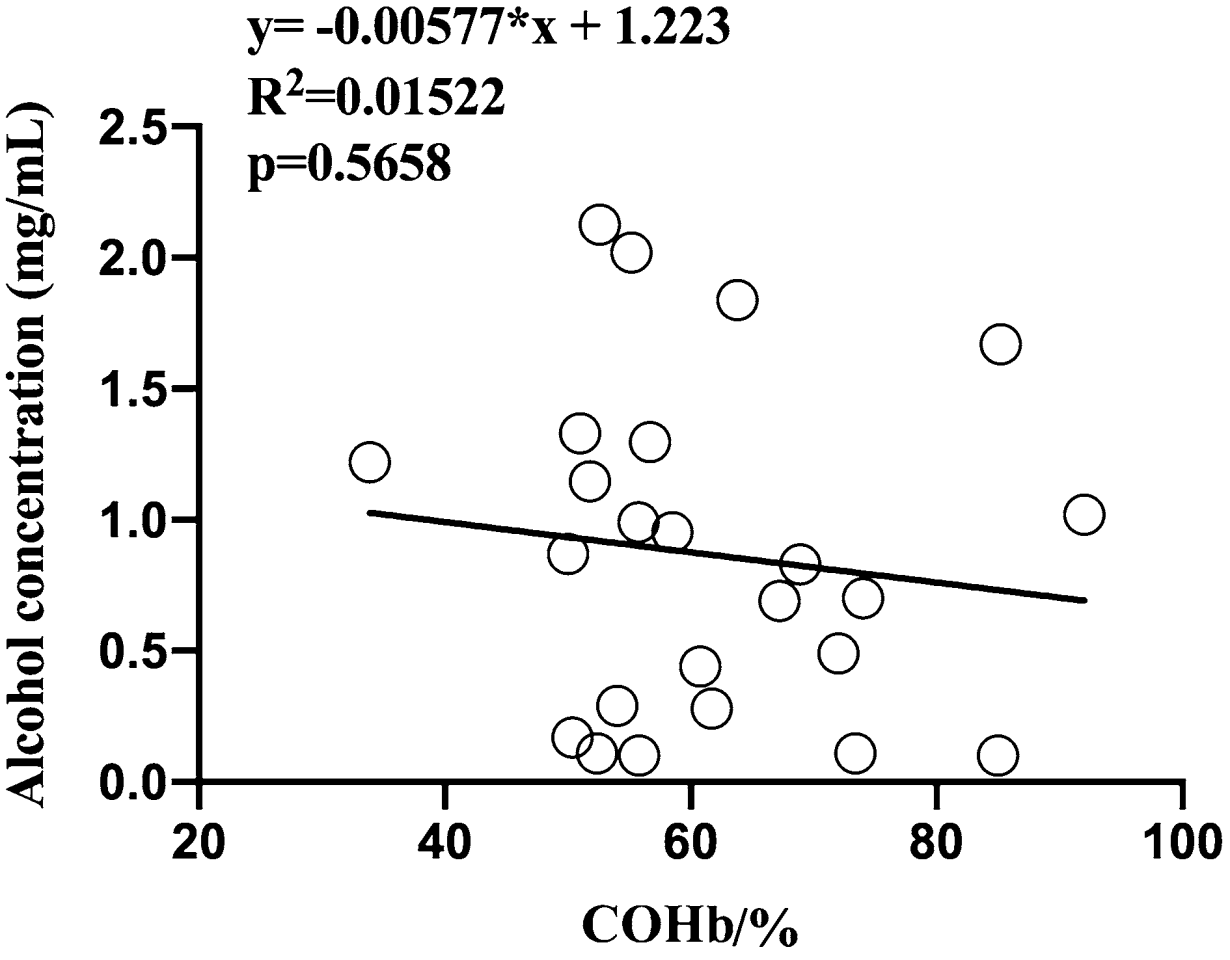
Correlation between COHb saturation and blood alcohol concentration in the fire-unrelated poisoning case.

Since fire was an important factor affecting the blood COHb% as observed above, we then divided cases into fire-unrelated and fire-related ones. In the fire-unrelated CO poisoning cases (n = 172 cases), the presence of alcohol was associated with a high COHb% (*p* = 0.023, Fig. 3a), which contradicted with psychotropic drugs that failed to significantly associate with the COHb% (*p* = 0.22, Fig. 3a). The COHb% in each subgroup did not significantly differ with regard to the toxicology (Fig. 3b). In the fire-related cases (n = 135 cases), alcohol or other psychotropic drugs did not significantly correlated with the COHb% neither in the whole cases (Fig. 3c) nor within each subgroup (Fig. 3d). Of note, though the presence of alcohol associated with higher blood COHb% in fire-unrelated poisoning cases (Fig. 3a), they did not correlate in a well linear manner (R^2^ = 0.015, *p* = 0.566, Fig. 4).

### Extremely low COHb%-related poisoning cases

In consistent with Table 1 that summarized the reported low COHb%-related CO poisoning cases, we found that some of the CO poisoning deaths were caused by extremely low COHb% (< 30%). These cases had explainable source of CO production, mildly increased COHb% (< 30%), and signs of CO poisoning, fulfilling the diagnosis of acute CO poisoning. In particular, 5 cases were detected with increased COHb% (mean COHb% = 24.44%) and negative of common psychotropic substances (Table 6). These cases were non-fire related, excluding the contribution of fire burn-related traumatic shock or hypovolemic shock. The 5 cases were also not decomposed when found, ruling out the effect of corpse condition on the detection of COHb%. In addition, medical examination excluded fatal injury, notable signs of asphyxia (i.e. pinpoint petechiae over skins and surfaces of organs), and cardiopulmonary pathology. Three of the 5 cases were females and younger than 30 years. All the 5 cases were found in an enclosed space. Scene investigation revealed 3 were accidental and the other 2 were suicidal.

**Table 6:** Details of the 5 deaths with low COHb saturation and negative pathological findings.

Case no	Gender	Age,years	Month	Manner of death	COHb%	Scene investigation	Medical examination
1	F	28	1	Accident	27.80%	The decedent was taking a shower in the afternoon while her workmate found her unresponsive on the floor of bathroom at evening	Non-specific
2	M	46	3	Suicide	21.00%	The decedent had disputes with and attacked his wife in the early morning. After taking his wife to hospital, the decedent was found dead at home in the morning	Non-specific
3	M	53	5	Suicide	26.00%	The decedent killed one person at his residence and was found dead at scene	Non-specific
4	F	6	8	Accident	23.60%	The decedent was found dead at home in a gas leakage incidence	Non-specific
5	F	22	12	Accident	23.80%	The decedent was found dead lying on the floor after forcing open the door by property manager	Non-specific

*Being non-specific denotes as no injury or macroscopic/microscopic pathology.

## Discussion

The present study investigated the characteristics of low COHb%-related CO poisoning deaths. We selected SPSB, the official institute that handles complicated and acute deaths in Shanghai, in order to collect sufficient cases of low COHb%-related poisoning cases. To avoid false negativity, some known factors that could affect the detection of COHb% were excluded such as putrefaction and delayed detection after blood collection. Persistent CO poisoning death (over 24 h), acute CO poisoning death with medical interventions, and death from complications of CO poisoning were also excluded. We also ensured that heart blood was immediately collected after notification of death and sent for laboratory detection with hermetic seal. COHb% was promptly detected and results were accessed within 30 min. In addition, the half-life of COHb in cadaveric bodies might also influence the detection results of COHb%. Due to substantial proportion of unwitnessed cases, we could not exactly determine the interval from exposure of CO to death. However, based on our knowledge, the half-life of COHb is approximately 250 to 320 min under room air environment in survivors who have experienced CO poisoning. The COHb% of a blood sample with an initial COHb% level of 70% could decrease to half at 20℃ after as long as 30 days without hermetic seal^31^. In the present study, though it’s difficult to determine the exact interval from exposure of CO to death and postmortem interval, the values for all cases were coarsely estimated to be less than 4 h which is far less than the half-life of COHb in living body and in corpse. Moreover, 170 of the 307 cases (55.4%) had a COHb% over 50%, while other cases referred to the same institute and occurred at the same period were detected of a COHb% less than 50%. Because all cases were selected from the same institute in a similar manner, no specific bias should have been introduced and thus the low COHb% should be indeed real instead of being due to postmortem artifacts. Therefore, rigorous inclusion criterion mentioned above ensured that the blood COHb% results were accurate and of minimal postmortem artifact. .

A total of 307 cases were retrieved and our data initially showed a decline trend for CO poisoning deaths over time. This tendency was consistent with previous results from the United States, England and Wales^32–34^ but opposite to some year-matched studies in the mainland China^35–37^. In a six-year (2009–2014) epidemiological study for CO poisoning deaths in Wuhan, China, the incidence rate of CO poisoning remained relatively stable over years^35^. In Liaoning, a northeast province from China, the annual number of poisoning deaths increased gradually from 8 cases in 2008 to 21 cases in 2017^36^. This inconsistency might be explained by regional differences in China.

Among the 307 CO poisoning deaths, group 1 (COHb% < 30%) claimed 58 cases (18.9%) and group 2 (30 ≤ COHb% < 50%) claimed 79 cases (25.7%). High COHb% levels (group 3) were only observed in 170 deaths (55.4%). The high frequency of low COHb%-related CO poisoning might be due to the case sources. As stated in the setting, the SPSB is an official organization that handles complicated cases. Common and undisputed poisoning cases are processed by the public security branches at each district of Shanghai city. However, this selection of case source guaranteed retrieval of sufficient cases that could serve analysis of this study. We found that CO poisoning deaths with low blood COHb levels had several features. First, winter was the high-incidence season, which was consistent with a previous study that reported a significant increase of poisoning in the fall-winter period^38^. Second, the low COHb%-related poisoning could occur in all ages of people. In group 1, each age group showed a relatively equal rate of deaths (~ 20%) which contradicted with group 2 and group 3 that had the vast majority of deaths in younger than 30 years, followed by 31–45 years. Our finding might indicate low COHb%-related CO poisoning had no age preference.

Interestingly, the present study found a strong association of low COHb% with a fire circumstance. This was basically consistent with previous publications as illustrated in Table 1, where 2 of the 3 fire-related CO poisoning deaths were associated with extremely low COHb% (20.70% and 10%, respectively), and the 7 non-fire related cases (gas leakage) were with COHb% ranging from 20.0% to 44.40% (Table 1). Previously, the effect of fire burn on the blood COHb% seemed to be controversial. A study from Portugal collected 69 samples and concluded that COHb saturation was lower in thermal injured decedents^16^, while another study from the United States examined 87 victims in a fire accident and found the vast majority (97%) of the decedents had a COHb saturation over 50%, with an average COHb saturation value of 76.5%^39^. Using the cases over 18 years, we concluded that fire-related CO poisoning presented with significantly decreased COHb levels. Several possible explanations could be conceived to interpret the negative effect of fire burns on COHb%. (1) Decedents found in fire scene had inhaled not only CO, but also other poisonous gases generated in combustion such as hydrogen cyanide (HCN) and sulfur dioxide (H_2_S)^39–43^. In a previous study, blood samples in 169 of 285 (59%) fire-related deaths were examined to be HCN positive with an average concentration of 16.83 mg/L, and half of the survivors were proved to be HCN positive (average 4.0 mg/L)^44^. These poisonous gases might intensify the acute CO poisoning and hasten the death. In a 3-year study of blood samples from 61 cadavers in fire-related cases, a total of 39 types of compounds were detected, including aliphatic hydrocarbons and aromatic hydrocarbons^45^. However, the method for detecting other poisonous gases remains restricted. These gases always failed to be screened in routine practice since they were “unknown” gases. So far CO is a quantifiable toxic gas which can be detected with higher accuracy in forensic toxicology; therefore, CO poisoning is usually allocated as the cause of death when the history of exposure is confirmed. Hence we might overestimate the effect of CO in fire-related poisoning and it is mandated to build up accurate methods screening of other common gases in suspected CO poisoning, especially when CO alone could not explain the death^46^. (2) Most victims in fire-related cases suffered from skin injury, which could aggravate CO poisoning. A previous study revealed that more than 50% victims in fire scene experienced burn shock^42^. After damage, the skin is not protective enough for the muscular tissue, leading to easy binding of CO to skeletal muscle, forming the carbonyl myoglobin that worsens anoxia. Fire-related cases also frequently presented with thermal injury and smoke inhalation which could cause spasms of the upper airway^47^. The thermal spasms of upper airway might prevent from further CO inhalation, making asphyxia as a contributing factor to the CO poisoning. (3) Alcohol might contribute to CO poisoning. We found that it was alcohol, but not other psychotropic drugs, that significantly associated with the blood COHb levels. It has been reported that alcohol did not have a direct interaction with CO, but it might relate to the impairment of organs during the intoxication^48^. Alcohol intake is also a high-risk factor to make decedents loss of consciousness and thereby leading to accelerated deaths in fire scenes^48–50^. (4) Potential cardiopulmonary diseases also contributed to CO poisoning with low COHb%^7^.

In addition, in the fire-unrelated CO poisoning cases, a combination with alcohol, but not psychotropic drugs, significantly elevated the blood COHb% levels as compared with mere CO poisoning cases. However, no significant linear correlation between COHb saturation and alcohol concentration was observed. This finding was consistent with the result of another study which examined 131 cases and concluded that there was no significant difference regarding COHb saturation between individuals with or without ethanol in blood^49^. This might reinforce the notion that alcohol unlikely directly interacts with COHb in blood but probably causes general damage to tissues and organs, hence affecting COHb% in an indirect way.

It is worth noting that even after systemic examination, there were still 5 cases that were low COHb%-related CO poisoning. These deaths were excluded from fire burn-related causes, mechanical injury, or notable signs of asphyxia. Systemic evaluation revealed source of CO production, mildly increased COHb% (< 30%), and signs of CO poisoning (i.e. cherry-red livor mortis or cyanosis of lips/fingernails), which fulfilled the diagnosis of acute CO poisoning. However, no significant cardiopulmonary pathology was revealed, and no combined psychoactive substances were detected, indicating there might be other inner mechanisms that mediates CO poisoning. Our observation was similar with previous reports, where 15 out of the 18 cases (83.3%) were free of psychoactive substances and 77.8% of deaths were not recorded of cardiopulmonary pathology (Table 1). In view of these intricate cases, it is highly suggested that more diagnostic markers, in addition to COHb saturation, should be examined. For a long period, the diagnosis of CO poisoning relies on the detection of high COHb%. However, in circumstances of low COHb%-related CO poisoning, it always causes disputes due to the unconvincing COHb%. COHb% should not be the mere reliable index in such circumstances since COHb% detection could be negatively affected by multiple factors^12,51,52^. To address this dilemma, molecular markers might represent an assistant diagnostic technique. The fibronectin and C5b-9, for example, have been found to be highly expressed in the myocardium in cases of CO poisoning^53^. The expression of heme oxygenase-1(HO-1) in brain tissue was reported to be activated in CO poisoning, in response to oxidative stress induced by hypoxia in rat CO poisoning^54^. An induced expression of HO-1 alleviated hippocampal damage after CO exposure, suggesting that HO-1 could be an appropriate marker for diagnosis of CO poisoning^55^. Therefore, the surge for sensitive molecular evidences is mandatory to aid in the diagnosis of low COHb%-related poisoning.

Finally, this study is subject to limitations that not all cases underwent full histological examination so the effect of any potential lung or heart diseases on COHb% could not be assessed. The exact duration from exposure of CO to death is unknown due to substantially unwitnessed deaths and whether this duration affects the detection of blood HbCO% merits future investigation.

## Conclusions

As compared with high COHb%-related CO poisoning, low COHb%-related CO poisoning could be observed in any age group and mostly in the winter season. Fire burns and the consumption of alcohol, but not other psychoactive substances, significantly associated with lower COHb%. In conditions of extremely low COHb%-related CO poisoning, further molecular markers are mandated to be identified in order to avoid disputes.

## Ethical standards

This article does not contain any studies with human participants or animals performed by any of the authors.
